# Hypothalamic obesity

**DOI:** 10.1007/s11154-026-10050-9

**Published:** 2026-05-28

**Authors:** Ismaa Sadaf Farooqi, Stephen O’Rahilly

**Affiliations:** Institute of Metabolic Science, University of Cambridge Metabolic Research Laboratories, Addenbrooke’s Hospital, Cambridge, UK

**Keywords:** Obesity, Hypothalamus, Leptin, Melanocortin

## Abstract

Hypothalamic circuits play a critical role in the regulation of energy homeostasis, a function that is essential for survival. Lesions of the hypothalamus in animals can cause weight gain or weight loss depending on their precise anatomical location and the chemical nature of circuits which are disrupted. Here, we discuss how human genetic disorders which impair the function of key molecular components of these hypothalamic circuits cause hyperphagia and obesity. The detailed molecular and physiological characterisation of these disorders provides direct insights into the mechanisms by which hypothalamic circuits and their projections regulate human physiology and behaviour. Increasingly, the finding of a genetic cause for a person’s obesity can inform clinical care and the use of targeted therapies.

Clinical trial number: not applicable.

## Hypothalamic regulation of energy homeostasis

The hypothalamus is an ancient, subcortical brain region present in all vertebrates whose primary function is to maintain a constant internal environment by adapting to changes in the external environment – the concept of homeostasis first described by Claude Bernard (1849) and Walter Cannon (1926). Within the hypothalamus, distinct clusters of neurons, organised within anatomically defined regions or nuclei, detect and integrate changes in sensory signals from the external and internal environment, compare these inputs to basic set-points for nutritional state, body temperature, thirst and other parameters, then activate autonomic, endocrine and behavioural outputs to restore physiological set-points [1].

**Fig. 1:**
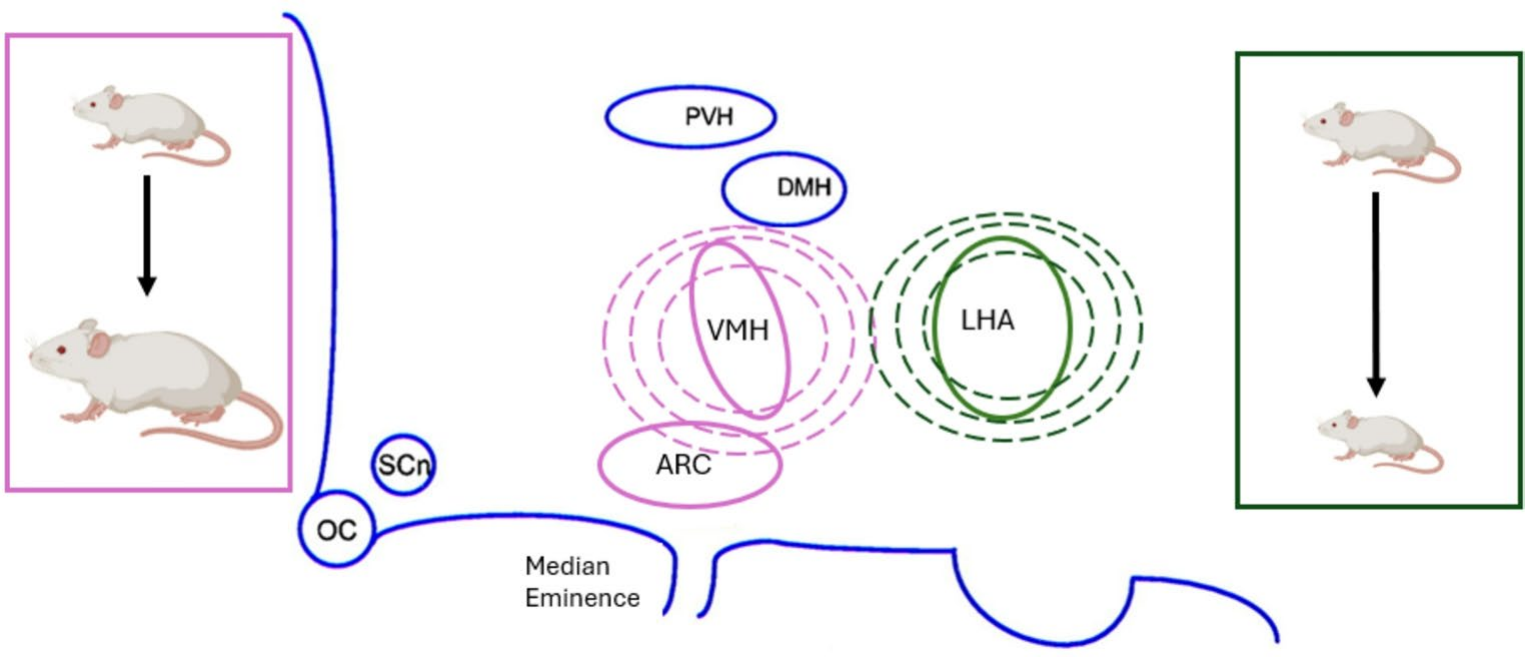
Disruption of key hypothalamic nuclei impacts body weight in mice. A schematic of the hypothalamus, indicating how surgical/chemical/electrolytic lesions involving the ARC (arcuate nucleus) and/or VMH (ventromedial hypothalamus) cause hyperphagia and obesity in mice, whereas lesions affecting the LHA (lateral hypothalamic area) cause hypophagia and weight loss. Other key regions involved in energy homeostasis, DMH (dorsomedial hypothalamus), PVH (paraventricular hypothalamus), are shown alongside key anatomical landmarks, the optic chiasm (OC) and suprachiasmatic nucleus (SCn)

The concept of energy balance/homeostasis was first described by Kennedy who showed that rats respond to acute caloric restriction by rapidly increasing food intake to restore their weight trajectory, allowing him to propose that circulating signals inform the brain regarding the amount of body fuel stored in the form of fat [2]. In the early 1900s, Brobeck and Frohlich reported children with tumors involving hypothalamo-pituitary structures associated with hyperphagia (increased drive to eat) and obesity, findings which suggested that the hypothalamus plays a key role in maintaining energy homeostasis. Subsequently, chemical and electrolytic lesioning experiments in rodents and cats in the 1930s and 1940s established how specific clusters of neurons (nuclei) within the hypothalamus disrupt energy balance, by changing energy intake and/or expenditure [3–5]. The impact of these lesions on weight gain/weight loss was partly determined by the size and precise anatomical location of the lesions, which suggested there were specific hypothalamic circuits that promote or suppress feeding behavior (Fig. 1) [3,6–8]. Subsequent studies showed that the proposed set-point for body weight can differ between lean and obese animals [9]. Speakman and colleagues have proposed that rather than a single value for a set-point, a set-point range within which people and animals oscillate depending on their environment, may be a more appropriate framework [10].

## Parabiosis and the discovery of leptin

Pioneering experiments by Hervey and Coleman used the technique of parabiosis, where the circulatory system of two animals is connected surgically allowing blood-borne factors to pass between them. In severely obese inbred strains of mice (*ob/ob* and *db/db*) [11, 12], Coleman demonstrated that *ob/ob* mice were missing a circulating factor which was present in excess in *db/db* mice and to which they were unresponsive. The identity of that circulating factor was finally revealed in 1994, by Jeff Friedman and colleagues who positionally cloned the *ob* gene and characterised its protein product, the hormone leptin [13].

Leptin, a hormone made by adipocytes, circulates in the blood in proportion to the amount of body fat stores. Leptin deficiency in *ob/ob* mice causes profound hyperphagia (increased drive to eat) and obesity, which is reversed upon leptin administration [13–16]. Ahima and Flier showed that leptin’s primary role is to defend against starvation. In mice, acute caloric restriction results in a fall in leptin levels which causes rebound hyperphagia and increases food consumption to restore energy homeostasis [17]. Leptin signals through the long isoform of the leptin receptor, which is widely expressed in the hypothalamus and other brain regions involved in energy homeostasis [18]. Leptin acts on primary melanocortin neurons in the arcuate nucleus (ARC) of the hypothalamus, an area with a relatively permeable blood-brain barrier [19], to increase food intake in states of nutritional deficiency via Agouti-related peptide (AGRP) neurons or reduce food intake in the fed state via Pro-opiomelanocortin (POMC) neurons [20]. These primary ARC neurons project to the paraventricular nucleus of the hypothalamus (PVH), where they synapse with MC4R (melanocortin 4 receptor) expressing neurons whose activation reduces food intake [21]. In mice and humans, genetic disruption of the development and/or function of the leptin-melanocortin pathway increases the drive to eat (hunger), reduces satiety, increases food reward and increases the preference for dietary fat, behaviours which favour the consumption and storage of nutrients for survival [6]. Importantly, this circuit does not act in isolation: MC4R neurons in the PVH also regulate the hypothalamic-pituitary-adrenal (HPA) axis and synapse with preganglionic sympathetic neurons, mediating the stress response, regulation of blood pressure and substrate utilisation by the liver and adipose tissue [22, 23]. In addition, leptin-responsive neurons connect to neurons expressing Gonadotrophin Releasing Hormone, Kisspeptin and Oxytocin, coupling nutritional state to the control of the onset of puberty, reproductive behaviour, fertility and parental care [24]. Additionally, extra-hypothalamic projections to the limbic system, amygdala, hippocampus, periaqueductal grey (PAG) and other brain regions, convey information about the fasted/fed state to neural circuits which regulate emotion, socialisation, fear, memory and aggression [25, 26].

## Human monogenic disorders

Severe obesity can result from rare (less than 1% minor allele frequency) penetrant genetic variants which disrupt components of the leptin-melanocortin pathway. People with these genetic disorders experience an intense drive to eat (hunger), find food to be highly rewarding and have impaired fullness (satiety) leading to hyperphagia (increased food intake) and weight gain from early childhood. Whilst these disorders are rare, cumulatively up to 20% of children with severe obesity have chromosomal abnormalities or other penetrant rare variants that drive their obesity.

## Disorders of leptin production and action

Complete deficiency of the hormone leptin and its receptor causes severe hyperphagia, early-onset of obesity (from the 1st year of life), impaired immunity and hypogonadotropic hypogonadism [26]. Homozygous mutations in the gene encoding leptin result in a failure to produce any circulating hormone (undetectable leptin levels [26]), the production of a form of leptin which is detectable in the serum but bio-inactive [27] or very rarely, a mutant form of leptin which antagonises the leptin receptor [28]. While measurable changes in resting metabolic rate or total energy expenditure have not been demonstrated, abnormalities of sympathetic nerve function suggest impaired fat oxidation may contribute to the excess accumulation of body fat (55–60% of total body weight [29]). Children with leptin deficiency have abnormalities of T cell number and function [29], consistent with high rates of childhood infection and a high childhood mortality from infection particularly in environments where infectious diseases are prevalent [30].

Bi-allelic mutations in the gene encoding the leptin receptor (*LEPR*) are more frequent, being found in 2–3% of severely obese children from consanguineous families and have been identified in non-consanguineous families, where both parents carried rare heterozygous variants [31]. Serum leptin concentrations are commonly appropriate for the degree of obesity, however, in some instances, particular *LEPR* mutations result in abnormal cleavage of the extracellular domain of LEPR (which acts as a leptin binding protein), are associated with markedly elevated leptin levels. Leptin and leptin receptor deficiency are associated with hyperinsulinemia consistent with the severity of obesity and hypothalamic (secondary) hypothyroidism characterized by low free thyroxine levels and inappropriately normal or high-normal levels of serum thyroid stimulating hormone (TSH [32]). Typically, pubertal development does not occur with biochemical evidence of hypogonadotropic hypogonadism. However, there is some evidence for the delayed but spontaneous onset of menses in a small number of leptin and leptin receptor deficient adults [32]. Linear growth is appropriate in childhood, but in the absence of a pubertal growth spurt, final height is frequently reduced.

Congenital leptin deficiency is treatable with daily subcutaneous injections of recombinant human leptin which reduce hyperphagia, fat mass and hyperinsulinemia, reverse immune defects *invitro*, and permit the timely development of puberty [33]. Treatment is currently available to patients on a compassionate use basis in some countries or, in the UK, through a structured national commissioning process for ultra rare diseases. Some of the effects of LEPR deficiency are caused by impaired signalling through the downstream melanocortin 4 receptor. Setmelanotide, a second generation melanocortin receptor agonist, has been shown to be effective in this condition and is now licensed in many countries [34].

## Disorders of pro-opiomelanocortin production and processing

Homozygous or compound heterozygous mutations in *POMC* cause hyperphagia and early-onset obesity [35]. POMC is the precursor for adrenocorticotrophin (ACTH); accordingly, POMC deficiency often presents in neonatal life with features of ACTH/cortisol deficiency such as hypoglycaemia and cholestatic jaundice, requiring long-term corticosteroid replacement therapy. POMC deficient people have pale skin and red or light-coloured hair, due to the lack of melanocortin signaling at melanocortin 1 receptors in the skin. POMC deficiency may also impair the timing of puberty, effects which appear to be mediated by the Melanocortin 3 receptor (MC3R [36]). Hyperphagia and obesity in complete POMC deficiency can be entirely treated with a melanocortin receptor agonist (Setmelanotide), which is now licensed for children from the age of 2 years [37]. Heterozygous missense mutations affecting the function of POMC peptides and POMC processing have been described [38]; these variants significantly increase obesity risk but are not invariably associated with obesity. The potential efficacy of MC4R agonists in this group is currently being tested in clinical trials.

Prohormone convertase-1 (PCSK1, also known as PC1/3) is an enzyme that acts on proinsulin, proglucagon, and POMC and other peptides. Compound heterozygous or homozygous mutations in *PCSK1* cause neonatal small bowel enteropathy, glucocorticoid deficiency (secondary to ACTH deficiency), hypogonadotropic hypogonadism and postprandial hypoglycaemia due to impaired processing of proinsulin to insulin as well as severe, early onset obesity [39]. Elevated plasma levels of proinsulin and 32/33 split proinsulin in the context of low levels of mature insulin can be used to diagnose this disorder [40], which can be treated with Setmelanotide.

Rare penetrant variants in multiple genes can affect the development and/or function of POMC neurons. For example, variants in genes encoding the Semaphorin 3 ligands, receptors and co-receptors which direct the development of POMC neuronal projections are associated with early-onset obesity [41]. Heterozygous loss of function variants affecting Steroid receptor coactivator-1 (*SRC-1)* (which interacts with phosphorylated STAT3 [42]) and Pleckstrin-homology-domain interacting protein, *PHIP* which directly modulates POMC transcription [43], are associated with obesity and a range of other phenotypes [44]. Disruption of the membrane-expressed Serotonin 2c receptor, *HTR2C* which regulates the electrical activity of POMC neurons is associated with obesity, social anxiety and impaired memory [45]. Additionally, deletions affecting TRPC5, a nonselective cation channel which modulates the depolarization of POMC neurons, cause obesity, anxiety, autism (in males) and postnatal depression (in females [46]).

## Disorders of melanocortin 4 receptor mediated signaling

**Fig. 2:**
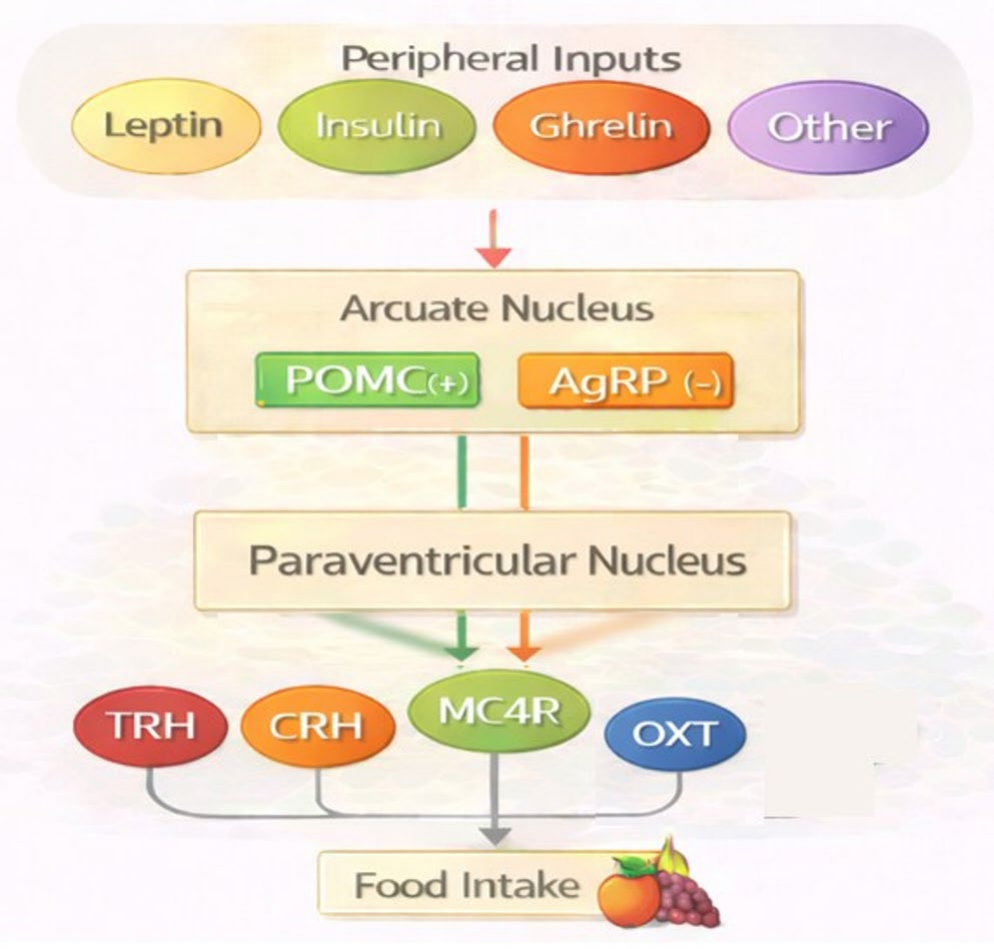
Overview of the leptin-melanocortin pathway whose disruption causes obesity in mice and humans. Primary neurons in the arcuate nucleus expressing POMC (pro-opiomelanocortin) and AgRP (agouti-related peptide), respond to peripheral hormonal signals as shown. In response, they either stimulate (+) or inhibit (-) the activity of second order neurons in the paraventricular nucleus expressing the melanocortin 4 receptor (MC4R). Activation of MC4R causes a reduction in food intake in the fed state, whereas its inhibition causes increased food intake in the fasted state. Disruption of other key neurons expressing Oxytocin (OXT), Corticotrophin-releasing hormone (CRH) and Thyroid releasing hormone (TRH) contribute to the phenotypic and behavioral spectrum seen in some genetic disorders affecting this pathway

POMC neurons project to second-order neurons in the paraventricular nucleus which express the Melanocortin 4 receptor (MC4R), whose activation reduces food intake in the fed state (Fig. 2). Heterozygous loss-of-function melanocortin 4 receptor (MC4R) mutations have been reported in people with obesity from various ethnic groups (www.mc4r.org.uk) and occur at a frequency of 1 in 300 people in the population [47], 1% of adults with a BMI > 30 kg/m^2^ and 3–5% of children with severe obesity. *MC4R* mutations are inherited in a co-dominant manner [48, 49], with variable penetrance and expression; homozygous mutations have also been reported. The clinical features of MC4R deficiency include hyperphagia, which often starts in the first few years of life. Alongside the increase in fat mass, MC4R-deficient subjects also have an increase in lean mass. They exhibit accelerated linear growth in early childhood, which may be a consequence of disproportionate early hyperinsulinemia and effects on pulsatile growth hormone (GH) secretion, which is retained in MC4R-deficient adults in contrast to common forms of obesity [50]. Reduced sympathetic nervous system activity in MC4R-deficient patients is likely to explain the lower prevalence of hypertension and lower systolic and diastolic blood pressures [51]. Thus, central melanocortin signalling appears to play an important role in the regulation of blood pressure and its coupling to changes in weight and to the regulation of lipid levels [52]. At present, there is no targeted therapy for MC4R deficiency, but patients with heterozygous *MC4R* mutations do respond to Glucagon-like peptide- (GLP-1) receptor agonists [53] and to Roux-en-Y-bypass surgery [54].

Heterozygous loss of function mutations in GNAS, the gene encoding Gas (which is imprinted), impair coupling to, or signaling by MC4R, which explains the hyperphagia and obesity seen when mutations are inherited on the maternal allele [55]. As Gas mediates signaling by multiple G-protein coupled receptors, variant carriers often develop hormone resistance and sometimes short stature (known as pseudohypoparathyroidism).

Homozygous mutations in ADCY3, the gene encoding adenylate cyclase 3, have been found in children with severe obesity [56]; heterozygous ADCY3 variants are associated with increased risk of obesity in people from Greenland [57]. Vaisse and colleagues showed that ADCY3 colocalizes with MC4R at the primary cilia of a subset of hypothalamic neurons and that inhibition of adenylyl cyclase signaling at the primary cilia of these neurons increases body weight, suggesting that impaired signaling from the primary cilia of MC4R neurons may contribute to human obesity [58]. Additionally, rare heterozygous variants in the melanocortin-2 receptor accessory protein 2 (MRAP2), a transmembrane protein that interacts with and regulates signaling by MC4R [59], are associated with obesity, with variable penetrance.

Rare penetrant variants in two transcription factors which control the development of the PVN are associated with obesity (Fig. 2). Chromosomal deletions and heterozygous loss of function mutations in Single minded 1 (SIM1) and Orthopedia (OTP) cause severe obesity [60, 61]. Clinical features of variant carriers resemble those seen in MC4R deficiency with, in addition, variable developmental delay with autistic features. Reduced oxytocin expression in the PVN of Sim1 haplo-insufficient mice and transgenic mice carrying loss of function human OTP variants may in part explain the behavioral features seen in humans [61].

## Ciliopathies

Primary cilium, antenna-like protrusions of the plasma membrane, on neurons throughout the brain sense external environmental signals and transduce intracellular signaling. Bardet-Biedl syndrome (BBS) and Alström syndrome arise from loss of function mutations affecting molecules which play a critical in the structural integrity of cilia and ciliary trafficking to cause obesity, retinal dystrophy, renal dysfunction, polydactyly and multiple other features. In mice, targeted genetic disruption of the development or function of cilia on hypothalamic Pomc neurons is sufficient to cause hyperphagia and obesity. Defects in leptin signaling and impaired ciliary localization of MC4R are mechanisms which are likely to contribute to obesity in these disorders. Clinical trials of Setmelanotide have shown some benefit in treating hyperphagia and obesity; this drug is licensed for BBS in some countries.

## Brain-derived neurotrophic factor signaling

Brain-derived neurotrophic factor (BDNF) signals via the Tropomycin-related kinase B (TrkB) and plays a key role in the development and maintenance of neurons, synapse formation and activity-dependent changes in synapse structure, throughput the brain. *Bdnf* and *TrkB* haplo-insufficient mice and mice in which *Bdnf* is deleted in the postnatal brain, exhibit increased food intake and weight gain. In humans, deletions encompassing the *BDNF* gene on chromosome 11p.12.3, a loss of function point variant in *BDNF* and several loss of function variants in *TrkB* have been reported in individuals with speech and language delay, hyperactivity, hyperphagia and severe obesity. Friedman and colleagues showed that a subpopulation of BDNF neurons in the ventromedial hypothalamus (VMH) act directly connect neurons in the Arc that receive interoceptive inputs (such as leptin) to premotor sites in the brainstem that regulate jaw movements and consummatory behaviour.

BDNF and TrkB expression is reduced in the hypothalami of people with Prader-Willi syndrome (PWS), an autosomal dominant disorder caused by deletion or disruption of a paternally imprinted region on chromosome 15q11.2-q12. Deletions encompassing a group of small nucleolar RNAs (HBII-85 snoRNAs) within the 4.5 Mb PWS region result in the cardinal features of PWS including obesity, suggesting that these snoRNAs play a critical role in the development of this syndrome. Histopathological studies on post-mortem brain samples from PWS patients have demonstrated reduced levels of oxytocin expression in the hypothalamus, a finding which may explain the neurobehavioral features seen in this condition; trials of intranasal administration in PWS are ongoing.

SH2B1 (Src homology 2B1) is an adaptor molecule involved in leptin, insulin and Brain-Derived Neurotrophic Factor (BDNF) signalling. Chromosomal deletions on 16p11.2 [62] and rare loss of function variants in the SH2B1 gene itself [63] are associated with hyperphagia, severe early-onset obesity, disproportionate insulin resistance and the early development of type 2 diabetes in adulthood [63]. Loss of function mutations in the SH2B1 gene are also associated with behavioural abnormalities such as aggression [63], a phenotype that is recapitulated in mice with neuronal deletion of Sh2b1 [64] and is likely mediated by impaired BDNF signaling. Obesity-associated mutations in SH2B1 have been shown to impair BDNF-mediated neurite outgrowth in cells, as such it is plausible that the obesity seen in SH2B1 variant carriers may be predominantly driven by their impact on BDNF, rather than leptin, signaling [65].

## Future perspectives

The characterisation of hypothalamic circuits involved in the control of energy homeostasis has shaped our understanding of the regulation of human appetite and body weight and had a direct impact on the diagnosis and treatment of people with severe obesity due to genetic disorders. Some of these treatments (e.g. the MC4R agonist Setmelanotide) have been shown to be effective in people with tumours which damage the hypothalamus and are now licensed for the treatment of obesity in children and adults with these debilitating conditions. Projections to and from the hypothalamus from the limbic system and brainstem are key to mediating food reward and meal-induced satiety. Effective therapeutic targeting of these circuits by the new generation of obesity medications is transforming the health of people with common obesity. A more comprehensive understanding of the physiological mechanisms which regulate neural circuits in the hypothalamus may improve understanding of the development of obesity, the challenges of weight regain and identify new targets for weight loss and weight maintenance.

## Data Availability

No datasets were generated or analysed during the current study.
